# Meta-transcriptomics Reveals Dysbiosis of the Respiratory Microbiome in Older Adults with Long COVID

**DOI:** 10.34133/research.0720

**Published:** 2025-06-02

**Authors:** Meng’en Liao, Jianpeng Cai, Feng Zhu, Yuanbo Lan, Tianqi Xu, Jingxin Guo, Quanlin Xue, Yilong Wen, Fan Zou, Yu Zhang, Shiliang Zhang, Yan Yan, Jingwen Ai, Jie Cui, Wenhong Zhang

**Affiliations:** ^1^ Shanghai Sci-Tech Inno Center for Infection & Immunity, National Medical Center for Infectious Diseases, Huashan Hospital, Institute of Infection and Health, Fudan University, Shanghai, China.; ^2^ Department of Respiratory and Critical Care Medicine, Affiliated Wuxi Fifth Hospital of Jiangnan University, The Fifth People’s Hospital of Wuxi, Wuxi, China.; ^3^ Department of Respiratory and Critical Care Medicine, Affiliated Hospital of Zunyi Medical University, Zunyi, China.; ^4^ Department of Respiratory, Wuxi Ninth Affiliated Hospital of Soochow University, Wuxi, China.; ^5^ Center of Clinical Laboratory, Affiliated Wuxi Fifth Hospital of Jiangnan University, The Fifth People’s Hospital of Wuxi, Wuxi, China.; ^6^ Shanghai Institute of Immunity and Infection, Chinese Academy of Sciences, Shanghai, China.; ^7^ Laboratory for Marine Biology and Biotechnology, Qingdao Marine Science and Technology Center, Qingdao, China.

## Abstract

Limited research has investigated the connection between long COVID (LC) and the respiratory microbiome, particularly in older adults. This study aimed to characterize the respiratory microbiome of older LC patients (with an average age of 65 years old), through meta-transcriptomic sequencing of 201 individual samples. Marked differences in microbial diversity were observed between LC and non-LC patients, including disruptions in both pathogenic bacteria and fungi. Importantly, viral taxa, such as *Herpes simplex virus type 1* and *Human coronavirus 229E*, were more frequently detected in LC patients, indicating the vulnerability of LC patients to viral infections. Functional annotation at the expression level revealed notable differences in microbial metabolism with alterations observed in pathways related to tryptophan–serotonin metabolism in LC patients. These findings underscore the altered microbial landscape, especially in older adults who developed LC, and fill the gap for the potentially clinical roles played by the respiratory microbiome.

## Introduction

The ongoing impact of coronavirus disease 2019 (COVID-19) has brought great attention to the long-lasting and often incapacitating sequelae that follow in the wake of severe acute respiratory syndrome coronavirus 2 (SARS-CoV-2) infection, a condition now formally recognized as “long COVID” (LC) [1,2]. Typically, among older adults, the impact of this condition not only increases mortality and hospitalization rates [3–5] but also results in an elevated susceptibility to more severe and persistent LC symptoms compared to those in younger individuals, further complicating recovery and leading to a more challenging postinfection course [6]. LC is a heterogeneous and multisystemic condition with a diverse array of symptoms—including persistent fatigue, cognitive impairment, breathing difficulties, gastrointestinal disturbances, autonomic dysfunction, and musculoskeletal issues—and mental health challenges [5]. It is widely acknowledged that older adults with LC symptoms face distinct and prolonged health threats [7,8], and therefore, understanding the pathophysiology related to LC is crucial.

The human respiratory tract, spanning from the nostrils to the lung alveoli, hosts niche-specific multikingdom microbial communities that likely act as gatekeepers against pathogens and support respiratory physiology and immune homeostasis [9,10]. As immune function declines with age [11,12], the respiratory microbiota undergoes significant changes [13,14], exemplified by the nasopharyngeal microbiome losing its niche specificity and becoming increasingly dominated by *Streptococcus*, *Prevotella*, and *Veillonella*, which may elevate the risk of infection [15]. Age-related microbial dysbiosis is linked to chronic inflammation, increased vulnerability to infections, and other respiratory diseases [16–21]. These issues are particularly relevant for LC patients. However, the dysregulation of key microbial taxa within the respiratory microbiome of elderly patients, particularly how these imbalances may exacerbate conditions associated with LC, remains a critical yet insufficiently explored area of investigation.

The human microbiome is intricately linked to LC. Emerging evidence based on animal models suggests that dysbiosis may have a causal role in LC [22]. LC patients exhibit gut microbiome dysbiosis characterized by a reduction in beneficial short-chain fatty acid-producing bacteria, such as *Faecalibacterium prausnitzii* [23–25] and *Bifidobacterium* [26,27], along with an increase in opportunistic pathogens like *Ruminococcus gnavus* [23,28,29] and *Flavonifractor* [27,30]. These microbial alterations correlate with systemic inflammation and immune dysregulation, potentially perpetuating chronic symptoms [31–34]. Specific LC symptoms have also been linked to gut microbiome changes; for instance, respiratory dysfunction is associated with an enrichment of *Veillonella* and *Flavonifractor* [30], while fatigue and neuropsychiatric symptoms correlate with *Clostridium innocuum* and *Actinomyces naeslundii* [23]. Given these connections, microbiome-based interventions, including probiotics [35–38], dietary modifications [39], and fecal microbiota transplantation [40], are being explored as potential therapies for mitigating LC symptoms.

Few studies have investigated the relationship between respiratory microbiota and LC. A study demonstrated that LC patients exhibited a markedly increased presence of pro-inflammatory and lipopolysaccharide-producing microbiota, including *Prevotella* and *Veillonella* [41]. Critical gaps remain in understanding the association between LC and the respiratory microbiome alteration, especially in older patients. Additionally, nonbacterial microorganisms, including viruses and fungi, which may also contribute to persistent LC symptoms, have been largely overlooked.

This study aims to investigate individuals’ respiratory-tract-resident microbiome, that is the collection of viruses, bacteria, and fungi, identified by unbiased total RNA sequencing, of middle-aged and older adults with LC symptoms and compare them to those of former patients who did not develop LC. We hypothesize that the respiratory microbiome alteration in LC patients is associated with the persistence of symptoms and could serve as a potential microbial biomarker for the condition. To fill such a gap, we performed unbiased meta-transcriptomic sequencing on throat swab and sputum samples, conducted cross-kingdom microbiome analyses, and explored functional changes in microbial communities and associations between microbiome composition and relevant clinical features.

## Results

### Subject characteristics and samples

**Fig. 1.**
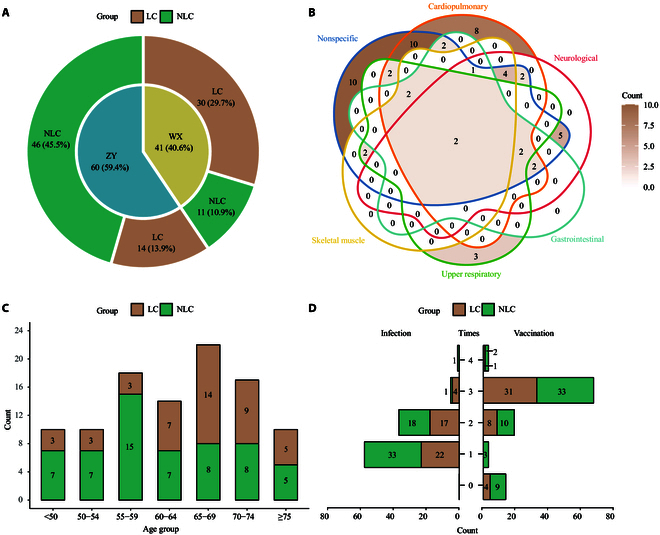
Overview of the study cohort and clinical characteristics. (A) Donut plot illustrating the proportion of long COVID (LC) and non-long COVID (NLC) patients across different centers (WX for Wuxi Fifth People’s Hospital and ZY for Affiliated Hospital of Zunyi Medical University). The inner circle represents patient recruitment sources, while the outer segments indicate the proportion of LC (brown) and NLC (green) patients within each center. (B) Venn diagram showing the distribution of clinical symptoms among LC patients, classified into 6 categories: cardiopulmonary, neurological, gastrointestinal, upper respiratory, skeletal/muscle, and nonspecific. Overlapping regions indicate the number of LC patients presenting with multiple categories of symptoms. (C) Age distribution of LC and NLC patients. Bars represent the count of patients in each age group, stratified by LC and NLC groups. The numbers of patients are shown. (D) Comparison of history severe acute respiratory syndrome coronavirus 2 (SARS-CoV-2) infection frequency (left) and COVID-19 vaccination status (right) between LC and NLC groups. Bars indicate the number of individuals in each group.

To investigate the changes in the respiratory tract microbiome of middle-aged and older adult patients with LC, we collected oropharyngeal swab (SW) and sputum (SP) samples from individuals with a history of at least one SARS-CoV-2 infection. The study center was not a major determinant of respiratory microbiome alterations in LC patients, as shown by principal coordinates analysis (Fig. S1). Demographic and clinical information was collected simultaneously by using a structured questionnaire (Table S1). A total of 122 participants completed the questionnaire, and 201 paired samples from 101 participants—comprising 101 sputum specimens and 100 throat swab specimens—were subsequently processed for sequencing and analysis. Of these participants, 43.6% (*n* = 44) reported experiencing persistent symptoms following COVID-19 infection (Fig. 1A). Nonspecific symptoms, such as sleep disorders and memory loss, along with cardiopulmonary symptoms including cough and difficulty breathing, are among the most frequently reported symptoms in patients with LC (Fig. 1B). Coexistence of multiple symptom types was commonly observed. The average age for patients with LC symptoms was 65.2 years, compared to 60.8 years for those without LC (Fig. 1C). Forty-one (40.6%) participants experienced multiple infections, and 88 (87.1%) participants received at least one COVID-19 vaccine (Fig. 1D). Overall, the cohort design and sampling strategies offer a valuable opportunity to investigate age-related changes in the respiratory tract microbiome specific to post-COVID-19 sequelae.

### Distinct taxonomic and diversity shifts in the respiratory microbiome of LC patients

**Fig. 2.**
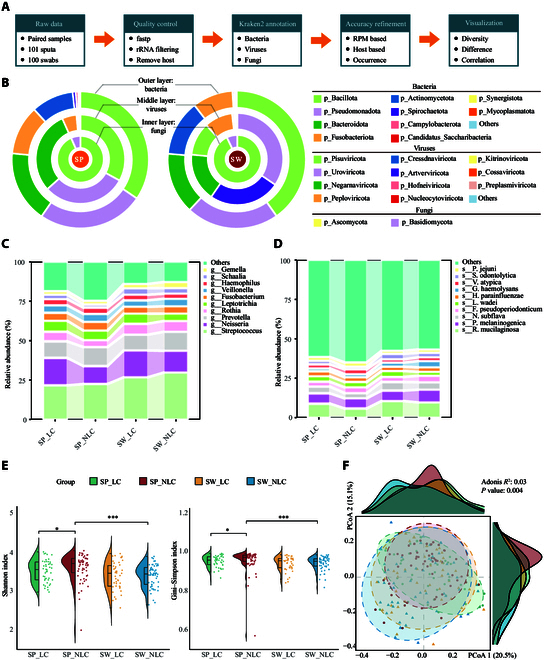
Microbiome profiling of upper and lower respiratory tract samples. (A) Schematic diagram of the analytical workflow for meta-transcriptomic data processing. (B) Phylum-level composition of the respiratory microbiome in sputum (SP) and swab (SW) samples. Donut plots depict the relative abundance of bacterial (outer ring), viral (middle ring), and fungal (inner ring) phyla. For clarity, the same color suite was used for different layers. Colors within each ring correspond to distinct phyla listed on the right. (C) Genus- and (D) species-level community composition presented by the relative abundance of bacterial taxa across 4 groups. The top 10 dominant taxa at each taxonomic level are highlighted. s__R. mucilaginosa, s__Rothia_mucilaginosa; s__P. melaninogenica, s__Prevotella_melaninogenica; s__N. subflava, s__Neisseria_subflava; s__F. pseudoperiodonticum, s__Fusobacterium_pseudoperiodonticum; s__L. wadei, s__Leptotrichia_wadei; s__H. parainfluenzae, s__Haemophilus_parainfluenzae; s__G. haemolysans, s__Gemella_haemolysans; s__V. atypica, s__Veillonella_atypica; s__S. odontolytica, s__Schaalia_odontolytica; s__P. jejuni, s__Prevotella_jejuni. (E) Violin plots showing the alpha diversity indices (Shannon index and Gini–Simpson index) for sputum (SP) and swab (SW) samples in 4 groups: SP_LC, SP_NLC, SW_LC, and SW_NLC. Statistical comparisons were performed using the Wilcoxon rank-sum test. (F) Principal coordinate analysis (PCoA) based on Bray–Curtis dissimilarity, illustrating beta diversity differences among the SP_LC, SP_NLC, SW_LC, and SW_NLC groups. Each point represents a sample, colored and shaped according to group. The variance explained by PCoA 1 and PCoA 2 is shown. Statistical significance was assessed using permutational multivariate analysis of variance (PERMANOVA). *P < 0.05; **P < 0.01; ***P < 0.001. rRNA, ribosomal RNA; RPM, reads per million.

To optimize the recovery of cross-kingdom microbiome composition, we analyzed the microbiomes of the upper respiratory tract (URT) and lower respiratory tract (LRT) in LC and non-long COVID (NLC) patients using unbiased meta-transcriptomic sequencing (Fig. 2A). After removing low-quality reads, human-genome-derived reads, and ribosomal RNA (rRNA) sequences, the clean reads were processed using the Kraken2 annotation workflow. To minimize false-positive hits, the following criteria were prioritized: (a) the confidence cutoff of Kraken2 was set as 0.1; (b) the reads per million (RPM) threshold was 10 for bacteria and 1 for fungi and viruses; (c) viruses with a known host of human, bacterial, or fungal origin were retained; and (d) bacterial and fungal microorganisms present in multiple samples were retained. Only microorganisms that met these requirements were included in the subsequent analysis. These strategies provided stringent characterization of respiratory tract microbiome for patients with a COVID-19 infection history. It was observed that URT and LRT exhibited similar bacterial compositions at the phylum level, with Bacillota, Pseudomonadota, and Bacteroidete*s* as the dominant phyla, along with fungal phyla Ascomycota and Basidiomycota (Fig. 2B). Notably, the viral communities differed significantly between these 2 niches. In SP samples, the top 4 viral phyla were *Pisuviricota*, *Uroviricota*, *Negarnaviricota*, and *Peploviricota*, whereas in SW samples, they were *Uroviricota*, *Artverviricota*, *Negarnaviricota*, and *Pisuviricota*.

To investigate the respiratory tract microbiome between LC and NLC groups in 2 niches (i.e., SP and SW samples), we profiled the family-level microbial composition of each individual (Fig. S2A). The analysis of microbial taxa distribution across different sample types and patient groups revealed similar patterns with distinct microbial signatures that may be associated with disease states. Specifically, most bacterial taxa can be detected in 4 groups (Fig. S2B). The family-level analysis revealed significant differences between LC and NLC groups, with Burkholderiaceae (bacteria), Pichiaceae (fungi), and *Redondoviridae* (viruses) enriched in LC across both sputum and swab samples (Fig. S3). In sputum, LC samples showed increased Microbacteriaceae and Aspergillaceae, while commensal families like Staphylococcaceae were depleted [42–44]. Swab samples exhibited similar dysbiosis, with Treponemataceae enriched and health-associated Gemellaceae depleted [45–48].

At the genus level, the respiratory microbiome composition showed distinct alterations across the 4 groups, with *Streptococcus* consistently dominating in all groups (Fig. 2C). Notable differences were observed in the relative abundances of *Neisseria*, *Prevotella*, and *Rothia*, between LC and NLC groups. Specifically, LC groups demonstrated a reduction in *Streptococcus* and *Prevotella*, alongside an increase in *Neisseria*, suggesting potential microbial dysbiosis associated with LC. *Rothia mucilaginosa* emerged as the most dominant species in all groups and showed higher relative abundance in LC patients than in NLC groups (Fig. 2D).

To identify the respiratory microbial signatures associated with LC, we conducted comparative analyses of community-level diversity. A significant decrease in alpha diversity, measured by the Shannon and Gini–Simpson indices, was observed in sputum samples from LC patients compared to those from NLC patients (*P* < 0.05, Wilcoxon test, Fig. 2E). In terms of sampling sites, LC patients showed comparable alpha diversity between the 2 sites (*P* > 0.05, Wilcoxon test, Fig. 2E). In contrast, NLC patients showed higher alpha diversity in sputum samples than swabs (*P* < 0.001, Wilcoxon test, Fig. 2E). Consistently, significant differences in microbial composition were observed between groups stratified by symptom status and sample types, as shown by the result of the permutational multivariate analysis of variance (ANOVA) test (Adonis *R*^2^ = 0.03, *P* = 0.004, Fig. 2F). However, these factors accounted for only a small portion of the total variation. The first principal coordinate (PCoA 1) explained 20.5% of the total variance observed, while the second principal coordinate (PCoA 2) accounted for 15.1% of the variance. Taken together, these results revealed altered microbial diversity associated with LC in older adults, especially in the LRT.

### Patients with LC showed dysregulated pathogenic microbes

**Fig. 3.**
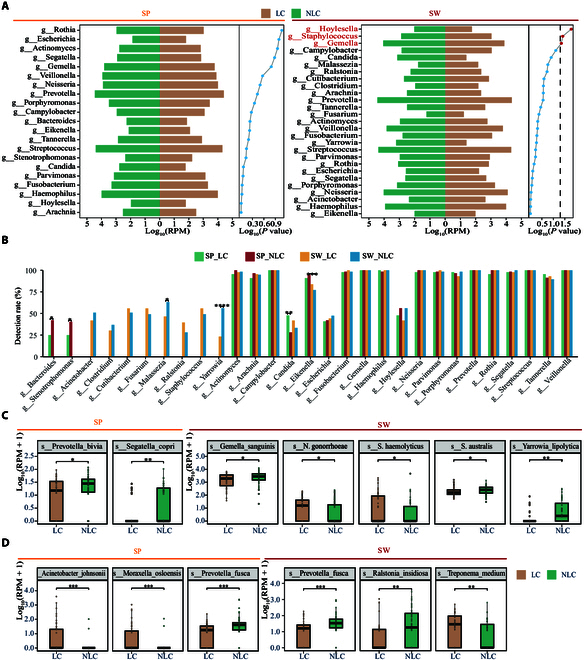
Differential analysis of pathogenic and nonpathogenic taxa between LC and NLC groups. (A) Bidirectional bar plots showing the genus-level relative abundance of pathogenic microbes annotated using the Global Catalogue of Pathogens. The left panel displays results for sputum (SP) samples, and the right panel for swab (SW) samples. Brown bars indicate LC groups, and green bars indicate NLC groups. Wilcoxon rank-sum test results are shown on the right side of each plot. (B) Detection rate (%) of genera containing pathogenic microorganisms between groups. Significant differences were assessed using the chi-square test, with *P* values indicated for each genus. (C) Boxplots comparing the log-transformed relative abundances calculated as log_10_(RPM + 1) of significantly different pathogenic species between LC and NLC groups in both sample types. (D) Boxplots showing nonpathogenic species with significant differences in relative abundance between LC and NLC in SP and SW samples. The top 3 species are shown in each sample type. Statistical tests were performed using the Wilcoxon rank-sum test. *P < 0.05; **P < 0.01; ***P < 0.001. See also Fig. S3.

To explore potential pathogenicity from infecting organisms, we compared the abundance and occurrence of pathogenic and nonpathogenic microbes identified in this cohort. This was achieved by incorporating the Global Catalogue of Pathogens, a comprehensive resource that provides strong evidence on human pathogens [49]. In sputum samples, 2 genera (*Bacteroides* and *Stenotrophomonas*) showed a decreased detection rate, although no pathogenic genera showed significant differences in terms of relative abundance (Fig. 3A and B). By contrast, a genus containing pathogenic fungi, *Candida*, was more frequently detected in the sputum of patients with LC symptoms. Specifically, 2 species from the family Prevotellaceae, *Prevotella bivia* and *Segatella copri*, had lower average abundance in LC sputum than in NLC (Fig. 3C). In swab samples, 3 detected bacterial genera, *Hoylesella*, *Staphylococcus*, and *Gemella*, showed significantly changed abundance between LC patients and NLC patients (Fig. 3A). Another 2 genera (i.e., *Malassezia* and *Yarrowia*) showed decreased detection rates in patients with LC compared to those in NLC patients (Fig. 3B). Consistently, most detected pathogens showed reduced relative abundance in LC patients, including *Gemella sanguinis*, *Streptococcus australis*, and *Yarrowia lipolytica* (Fig. 3C). Conversely, only *Neisseria gonorrhoeae* showed increased average abundance in these patients (Fig. 3C). We also identified significantly different nonpathogenic microbial species between LC and NLC patients (Fig. S4). The most differential microbes were *Acinetobacter johnsonii*, *Moraxella osloensis*, and *Prevotella fusca* in the LRT and *P. fusca*, *Ralstonia insidiosa*, and *Treponema medium* in the URT (Fig. 3D). Collectively, these results demonstrated a reduction in most differential pathogenic microorganisms in older patients with LC, as reflected in both relative abundance and detection rates. However, the findings necessitate validation in larger cohorts to ensure consistency and further investigation into its implications for LC and the potential underlying mechanisms.

### Relationship between viral detection and LC in older adults

**Fig. 4.**
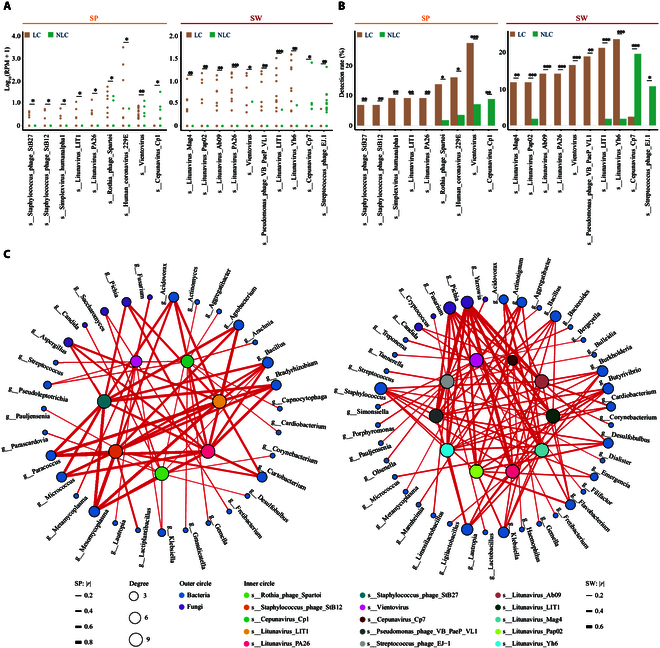
Comparative analysis of virus differences and host–microbe interactions in LC and NLC groups. (A) Boxplots showing the relative abundance calculated as log_10_(RPM + 1) of viral species with significant differences between LC and NLC groups in sputum (SP) and swab (SW) samples. Statistical significance was determined using the Wilcoxon rank-sum test. (B) Detection rates (%) of key viral species in SP and SW samples between LC and NLC groups. Chi-square test results are shown for each species. (C) Network analysis of virus–host interactions in SP (left) and SW (right) samples. Inner circles represent viral species with significant differences between LC and NLC groups, color coded by viral species. Outer circles display the top 30 bacterial (blue) and fungal (purple) genera that show significant positive correlations with these viruses. Circle size corresponds to interaction degree, and edge thickness represents the strength of correlation (Spearman correlation coefficient, |*r*| > 0.2). *P < 0.05; **P < 0.01; ***P < 0.001. See also Fig. S4.

Given the fact that SARS-CoV-2 interaction with human viral communities may serve as a potential biological contributor to post-acute sequelae of COVID-19 [50], we analyzed the species-level virome characteristics related to LC. Six viruses from the genus *Litunavirus* were detected in patients with LC (Fig. 4A and B), suggesting a possible connection between this genus of bacteriophage and LC symptoms. Among them, *Litunavirus LIT1* and *PA26* showed similar increasing trends in both sputum and swab samples, while the other 4 viruses, *Litunavirus Mag4*, *Pap02*, *Yh6*, and *Ab09* increased only in throat swabs. Similarly, several additional viruses were found to be uniquely present in LC patients, including *Simplexvirus humanalpha1* and *Staphylococcus phage StB12* and *StB27* in the LRT. *Simplexvirus humanalpha1* was known as *Herpes simplex virus type 1* (HSV-1) and has been associated with critically ill COVID-19 patients [51]. *Vientovirus* of genus *Torbevirus* showed an increased detection rate in the LRT and URT of LC patients. *Human coronavirus 229E* and *Rothia phage Spartoi* were detected in the sputum of both groups of patients with and without LC with a significant increase in LC patients. *Pseudomonas phage VB PaeP VL1* showed a similar trend in swabs. Several other viruses exhibited distinct patterns in their occurrence. *Cepunavirus* was not detected in LC patients; however, 2 of its members, *Cepunavirus Cp1* and *CP7*, were identified in 8.77% of sputum samples and 10.53% of swabs from NLC patients, respectively. *Streptococcus phage EJ-1* was much less frequently detected in LC patients. Collectively, most differentially detected viral species had increased detection rates in older patients undergoing LC, indicating that an exploratory study to investigate the underlying biological relationship is required.

A noteworthy human respiratory-associated virus was *Vientovirus* sp. (Fig. S5A and B), a circular Rep-encoding single-stranded DNA virus known to be elevated in the oro-respiratory samples of patients with periodontitis, acute illness, and severe COVID-19 [52]. Sequence analysis revealed that the *Vientovirus* genome contains a capsid protein, a replication-associated protein, and an open reading frame 3 protein (Fig. S5C), whose function remains unclear. The *Vientovirus* Rep protein (Fig. S5C) contains 2 domains found in many small DNA viruses: one involved in rolling-circle replication (InterPro: PS52020) and a second helicase domain within the P-loop NTPase superfamily (Pfam: PF00910) [53]. Based on the abundance difference of *Vientovirus* in patients with LC, the Rep protein of *Vientovirus* may serve as a potential biomarker for LC detection. *Human coronavirus 229E*, a human coronavirus typically associated with mild upper respiratory infections, may experience alterations in its prevalence and virulence potentially due to the influence of SARS-CoV-2 [54]. Considering the unique occurrence of *Human coronavirus 229E* in patients suffering from LC, we assembled contigs from meta-transcriptomic reads and identified a nearly complete genome of *Human coronavirus 229E* that showed 99.905% nucleotide similarity with *Human coronavirus 229E* strain LC22 (GenBank: ON791801.1) (Fig. S5D).

To link identified bacteriophages with possible host interactions, we conducted correlation network analyses in sputum and swab samples. The analysis in SP samples revealed strong correlations between specific bacteriophages and both bacterial and fungal genera (Fig. 4C, left panel). Notably, phages like *Litunavirus PA26* and *Staphylococcus phage STB27* showed extensive connections with multiple bacterial hosts, suggesting their potential role in modulating microbial dynamics. In the SW samples, the correlation network displayed a more intricate pattern, highlighting that phages such as *Pseudomonas phage VB PaeP VL1* and members in *Litunavirus* were prominently linked to genera like *Pichia* and *Fusarium* (Fig. 4C, right panel).

These findings suggested relatively differential viral members and phage–host interaction dynamics between the URT and the LRT, which may influence the persistence and symptomatology of LC.

### Functional potential differences in the expression of genetic materials of LC and NLC respiratory microbiomes

**Fig. 5.**
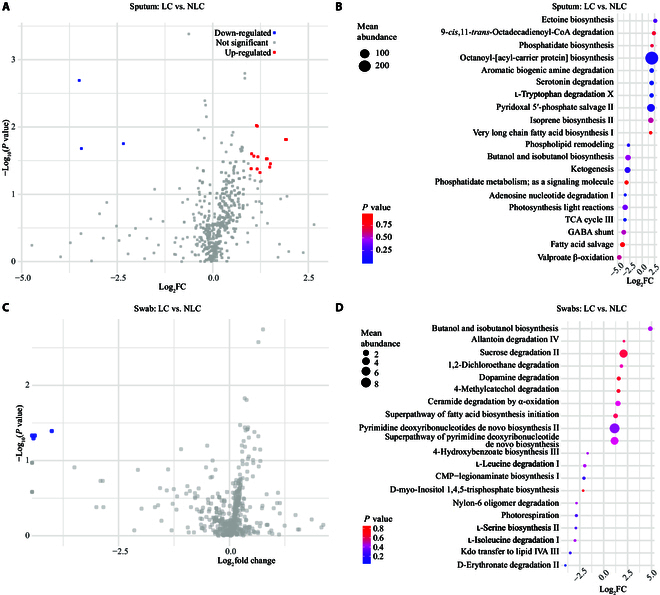
Differential abundance of respiratory microbial MetaCyc pathways between LC and NLC patients. (A) Volcano plot showing the differential MetaCyc pathway abundance between LC and NLC patients in sputum samples. (B) The top 10 LC associated and NLC associated MetaCyc pathways in sputum samples based on the Wilcoxon signed-rank test. The *y* axis lists the pathway names, and the *x* axis represents the log-transformed pathway abundance ratio (LC versus NLC). Bubble size corresponds to the mean abundance of the pathway in the respective group, while color indicates the *P* value. (C) Volcano plot showing the differential MetaCyc pathway abundance between LC and NLC patients in swab samples. (D) The top 10 LC associated and NLC associated MetaCyc pathways in swab samples based on the Wilcoxon signed-rank test. The *y* axis lists the pathway names, and the *x* axis represents the log-transformed pathway abundance ratio (LC versus NLC). Bubble size corresponds to the mean abundance of the pathway in the respective group, while color indicates the *P* value. FC, fold change; TCA, tricarboxylic acid; GABA, γ-aminobutyric acid; CMP, cytidine monophosphate.

One advantage of meta-transcriptomics is the capacity to analyze the activated genetic elements expressed by microbial communities. We analyzed the functional potential of the respiratory microbiome in LC and NLC patients using MetaCyc pathway data inferred through HUMAnN 3.0 [55]. Differential abundance analysis identified 26 MetaCyc pathways significantly associated with LC or NLC in sputum samples and 4 in swab samples (Fig. 5A and C). Pathways enriched in the NLC group included those involved in core metabolic functions, such as fatty acid salvage, adenosine nucleotide degradation, phospholipid metabolism, and amino acid metabolism (Fig. 5B and D). In LC patients, the most enriched pathway was ectoine biosynthesis, along with pathways related to aromatic biogenic amine degradation, serotonin degradation, and l-tryptophan degradation (Fig. 5B). These findings highlight distinct functional differences in microbial metabolism between LC and NLC groups.

### Association between the respiratory tract microbiome and clinical features

To explore the potential link between the respiratory tract microbiome and specific clinical features, we performed correlation analyses to identify significant microbial patterns. Firstly, the Mantel test was used to evaluate the correlation between microbial diversity at the family, genus, and species levels, and several factors, including age, gender, smoking status, vaccination, treatment, and the presence of LC symptoms (Fig. S6). The Mantel test revealed significant correlations between LC symptoms and clinical factors such as age, gender, and treatment status. These factors, especially at the species level, displayed stronger associations with microbial composition, consistent with our previous observations.

**Fig. 6.**
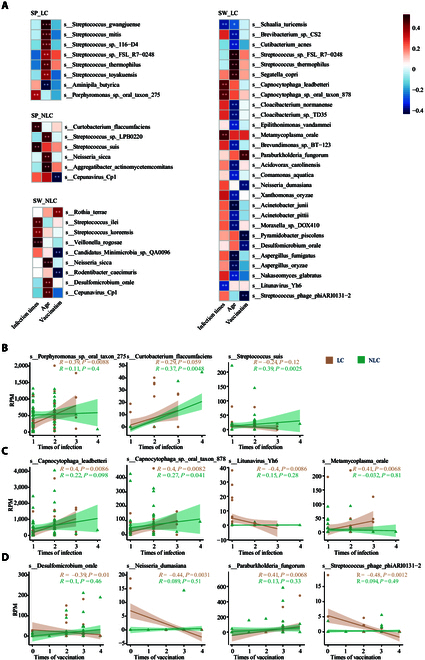
Correlation analysis between microbial species and clinical characteristics in LC and NLC groups. (A) Heatmap showing correlations between microbial species and 3 clinical characteristics, including infection times, age, and vaccination times, in SP and SW samples for LC and NLC groups. The top-ranked species were shown in each group. (B) Scatter plots showing the relationships between infection times and microbial species with significant correlations in SP samples. (C) Scatter plots showing the relationships between infection times and microbial species with significant correlations in SW samples. (D) Scatter plots showing the relationships between vaccination times and microbial species with significant correlations in SW samples. *P < 0.05; **P < 0.01; ***P < 0.001. See also Fig. S5.

To elucidate the correlations between specific microbial species and clinical parameters in middle-aged and elderly patients with LC, we conducted correlation analysis in sputum and swab samples from both LC and NLC groups (Fig. 6A). In SP samples, *Porphyromonas* sp. *oral taxon 275* and *Curtobacterium flaccumfaciens* exhibited positive correlations with infection frequency (Fig. 6A and B). Similarly, in SW samples, *Capnocytophaga leadbetteri* and *Metamycoplasma orale* showed positive correlations with infection times (Fig. 6A and C). Conversely, *Streptococcus suis* and *Litunavirus Yh6*, in the LRT and URT, respectively, negatively correlated with the times of previous SARS-CoV-2 infections (Fig. 6A to C). The correlation analysis between microbial species in swab samples from LC patients and the number of vaccinations revealed predominantly negative correlations, as seen in species like *Desulfomicrobium orale* and *Neisseria dumasiana* and the bacteriophage *Streptococcus phage phiARI0131-2* (Fig. 6A and D). Interestingly, *Paraburkholderia fungorum* exhibited a moderate positive correlation (*R* = 0.41, *P* = 0.0068) with vaccination frequency, contrasting with the general trend and indicating a particular response to vaccination among the analyzed microbes (Fig. 6A and D). Overall, these results revealed a complex interplay between microbial dynamics and clinical parameters, where samples from different respiratory sites exhibited heterogeneous microbial responses to infection frequency and vaccination.

## Discussion

LC remains a critical public health issue following the COVID-19 pandemic, particularly on older adults. While previous studies have examined gut microbiome changes in LC patients, the respiratory microbiome, especially in older adults, remains underexplored. This study addressed this gap by characterizing the respiratory microbiome of middle-aged and elderly LC patients using meta-transcriptomic sequencing of 201 individual samples. Our findings revealed microbial dysbiosis in LC patients, marked by reduced microbial diversity and disruptions in both pathogenic bacteria and fungi. Additionally, functional annotation at the expression level highlighted distinct alterations in microbial metabolism between LC and NLC groups, with LC patients showing enrichment in pathways related to ectoine biosynthesis, aromatic biogenic amine degradation, and l-tryptophan degradation. Importantly, LC patients exhibited increased susceptibility to viral infections, with higher abundances of viral taxa such as *Herpes simplex virus type 1*, *Human coronavirus 229E*, and *Vientovirus*. These metabolic shifts, alongside microbial and viral imbalances, suggest a complex interplay between bacteria, viruses, and fungi that could contribute to the prolonged symptoms observed in LC.

Previous studies have demonstrated reduced alpha and changed beta diversity of the gut microbiota in LC patients compared to those in NLC individuals, with an increase in opportunistic pathogens and a decrease in beneficial bacteria [56,57]. These findings align with our results, which also show reduced alpha diversity in the LRT. Some studies have suggested that opportunistic bacteria, such as members of the chronic-respiratory-condition-associated Burkholderiaceae family [58], are more enriched in COVID-19 patients [59]. At the genus level, significant increases in *Staphylococcus* [60,61] and *Gemella* [62] have been reported in both COVID-19 and LC patients, whereas *Bacteroides* and *Stenotrophomonas* showed a marked decrease [61,63]. Additionally, *Candida* has frequently been detected in COVID-19 intensive care unit patients and is often associated with severe infections [64]. At the species level, *P. bivia* has been negatively correlated with monocyte levels in COVID-19 patients [65], while the abundance of *Staphylococcus haemolyticus* has appeared to decrease [66]. Our study also observed similar variations in bacterial abundance or detection rates, suggesting that increased opportunistic pathogen levels may be associated with sustained immune dysregulation and chronic inflammation in LC patients [62,67,68]. Functionally, the enrichment of the tryptophan–serotonin metabolic pathway (e.g., aromatic biogenic amine degradation, serotonin degradation, and l-tryptophan degradation X) in the LC group suggests a functional implication in chronic inflammation and sleep disturbances, as serotonin plays a crucial role in modulating immune responses, reducing pro-inflammatory cytokines [69], and serving as a precursor for melatonin, which is essential for circadian rhythm regulation and sleep maintenance [70]. The highest enrichment of the ectoine biosynthesis pathway suggests a key role in anti-inflammatory protection and cellular stress resistance by respiratory microbiota.

The role of bacteriophages in human diseases remains underexplored. However, several studies suggest that bacteriophages may influence disease resistance or susceptibility through mechanisms independent of their bacterial hosts [71,72]. While adaptive phage therapy has been shown to reduce antibiotic use in the rehabilitation of post-COVID-19 patients in intensive care units [73], direct experimental studies investigating the contribution of bacteriophages to LC pathogenesis are needed. In our study, we found significant differences in bacteriophage abundance and detection rates between LC and NLC patients. These differences may be associated with bacterial hosts and the reactivation of latent infection following immunosuppression [74]. However, the specific role of bacteriophages in the progression of LC requires further investigation. A similar mechanism may explain the observed differences in the abundance and detection rates of respiratory viruses such as *HSV*, *Vientovirus*, and *Human coronavirus 229E*. HSV-1 reactivation may contribute to LC through immune modulation or indirect effects on the respiratory microbiome [75,76]. While *Vientovirus* is generally considered a commensal virus and does not appear to influence SARS-CoV-2 infection [77], its potential role in respiratory health and immune interaction in LC remains unclear and requires further investigation. Current studies on the relationship between redondovirus and SARS-CoV-2 remain inconclusive. Some research has reported higher abundance and detection rates of redondovirus in SARS-CoV-2-positive samples [78,79], while others found no significant association [80]. In our study, the differences in redondovirus abundance and detection between LC and NLC patients were notable but insufficient to establish a causal role in the progression of LC. Further research is needed to clarify the complex interactions between these viruses and the disease.

Our study identifies microbial and viral dysbiosis in older adults with LC, yet the cross-sectional design limits causal inference. While these imbalances may reflect associative consequences of post-COVID immune dysregulation, plausible mechanisms suggest bidirectional interactions. For example, HSV-1 [81] and *Human coronavirus 229E* [82,83] enrichment could drive chronic inflammation, while dysregulated commensals like *Gemella* may impair mucosal immunity [84,85], perpetuating dysbiosis. Longitudinal studies tracking microbial dynamics from acute infection to recovery, combined with mechanistic models, are needed to establish causality.

Several limitations of this study should be acknowledged. One limitation of this study is the absence of a healthy control group; however, prior studies have shown that the respiratory microbiome diversity in COVID-19-recovered patients without LC (NLC) tends to normalize toward healthy baselines [86–88], supporting our focus on the LC vs. NLC comparison to specifically identify microbial signatures associated with LC. Saliva contamination in throat swabs and sputum samples is difficult to eliminate entirely, but in this study, efforts were made to minimize this issue during sampling in this. While meta-transcriptomics offers a snapshot of cross-kingdom microbial activity, it lacks the genomic context of microorganisms with DNA as genetic materials needed to capture their full metabolic potential and complex interkingdom interactions. Future studies could benefit from integrating metagenomics to provide a more comprehensive view. Reliance on self-reported symptoms introduces potential recall bias and limits the precision of symptom characterization. Incorporating objective clinical measures, such as inflammatory markers (e.g., TIMP-1) that can be triggered by HSV-1 [89] and neurocognitive tests, could improve the accuracy of symptom–microbiome associations and help contextualize immune responses in LC. This study is cross-sectional in nature, which limits our ability to assess causal relationships and the temporal progression of microbial dysbiosis in LC. Future longitudinal studies are essential to better understand how microbial changes evolve over time and their role in the persistence of symptoms.

## Methods

### Study design and ethics approval

Participants in this study were patients with a history of at least one COVID-19 infection, recruited from 2 hospitals in China: 42 participants from Wuxi Fifth People’s Hospital with samples collected between 2023 November 19 and 2024 January 6 and 80 participants from the Affiliated Hospital of Zunyi Medical University with samples collected between 2024 January 20 and 2024 February 3. This cohort comprised individuals with real-time polymerase chain reaction-confirmed primary SARS-CoV-2 infection, followed up at a median of 12 months postdiagnosis. While subsequent reinfections were captured through self-reports, no virological data were available for these events. Symptoms and clinical characteristics were collected through a standardized questionnaire during visits. The follow-up was conducted in person with face-to-face visits between physicians and participants. The standardized questionnaire was validated through preliminary testing, where a small group of nonresearch individuals provided feedback, leading to adjustments in wording and format for clarity and comprehension. The questionnaire, described in our previous study [90], included details such as sex, age, smoking history, chronic disease history, number of COVID-19 infections, vaccination status, and prior treatments during infection.

Based on self-reported symptoms collected via questionnaires, 53 participants reported LC symptoms, such as persistent fatigue, respiratory difficulties, and cognitive impairments, and were assigned to the LC group. Individuals who were diagnosed with COVID-19 at the initial stage (SARS-CoV-2 positive) but did not report any LC symptoms during the follow-up period were referred to the NLC group (*n* = 65). The diagnosis of whether symptoms were related to LC was made by trained physicians. Ethical approval for this study was granted by the Ethics Committee of Wuxi Fifth People’s Hospital (approval no. 2022-019-1). All procedures performed were in accordance with the ethical standards of the committee. Written informed consent was obtained from all participants prior to enrollment in the study.

### Sample collection

Throat swabs were collected by trained personnel, and the swabs were then placed into the collection tube immediately. The method for induced sputum collection is as follows: subjects inhaled 4% sterile nebulized saline through a mouthpiece for 15 min. During the induction procedure, participants’ condition was assessed by the trained personnel. Subjects were instructed to rinse their mouths with clean water before sample collection and to spit saliva into a saliva container before coughing sputum into a sterilized sputum container. The inhalation period was interrupted every 2 min to allow subjects to cough up sputum. All samples are immediately placed in dry ice for storage and transported to the laboratory.

### RNA extraction and quality control

Total RNA extraction was performed using the RNA PowerSoil Total RNA Isolation Kit (MoBio, Cat. No. 12866-25), following the manufacturer’s protocols. To assess RNA quality, a 1.5% agarose gel electrophoresis was used, ensuring intact RNA bands without signs of degradation. RNA concentration and purity were measured using a NanoDrop NC2000 ultraviolet–visible spectrophotometer (Thermo Fisher Scientific, Waltham, MA, USA). Samples with an RNA concentration greater than 50 ng/μl and a total RNA quantity exceeding 1 μg were considered adequate for library preparation. RNA purity was confirmed by an *A*_260_/*A*_280_ ratio >1.8, although slight deviations were acceptable based on sample-specific characteristics. Additionally, RNA integrity number was assessed using an Agilent 2100 bioanalyzer, and samples with RNA integrity number ≥5.5 were used for further analyses.

### Library construction and sequencing

rRNA depletion was performed using rRNA-specific probes that hybridize to total RNA, followed by magnetic bead separation to remove rRNA/probe complexes. The remaining messenger RNA was further purified using ethanol precipitation. Complementary DNA (cDNA) libraries were constructed using the TruSeq Stranded mRNA LT Sample Prep Kit (Illumina, San Diego, CA, USA) according to the manufacturer’s protocol. Fragmentation of RNA was carried out using divalent cations under elevated temperature conditions (94 °C for 8 min). This was followed by first-strand cDNA synthesis using the Superscript III enzyme in the presence of Act D Mix, which was incubated at 25 °C for 10 min, 42 °C for 50 min, and then 85 °C for 15 min. Second-strand cDNA synthesis was performed using Second Strand Marking Master Mix, incubated at 16 °C for 1 h. Purified cDNA was recovered using AMPure XP beads. After second-strand synthesis, A-tailing was performed to add a single adenosine (A) base to the 3′ ends of cDNA fragments, preventing self-ligation. This was followed by the ligation of indexed adapters. Adapter-ligated cDNA fragments were amplified by polymerase chain reaction, followed by final purification and size selection using AMPure XP beads. Library quality was assessed using Agilent High Sensitivity DNA Kit on Agilent 2100 Bioanalyzer, where a single peak indicated proper library construction. Library quantification was performed using Quant-iT PicoGreen dsDNA Assay Kit on a Promega QuantiFluor instrument. Libraries with a concentration above 2 nM were deemed suitable for sequencing. Qualified libraries were sequenced on the Illumina NovaSeq 6000 platform at Personalbio (Shanghai, China), using NovaSeq 6000 S4 Reagent Kit (300 cycles) to generate paired-end reads of 2 × 150 bp.

### Sequencing data preprocessing

For the processing of raw sequencing reads, fastp (version 0.23.4) [91] was employed to filter out low-quality reads, including those that were too short or contained excessive ambiguous bases (N), as well as to trim low-quality bases, remove adapter sequences, and drop duplicated reads/pairs. Default parameters were used except for an accuracy level of 6 to calculate duplication. To remove rRNA sequences in silico, we used SortMeRNA (version 4.3.6) [92] with the smr_v4.3_default_db database built from the Silva 138 SSURef NR99 (16S, 18S), Silva 132 LSURef (23S, 28S), and RFAM v14.1 (5S, 5.8S) source databases with default parameters. The sequences were clustered at 90% identity for bacterial 16S rRNA, with 5S and 5.8S rRNA as seed sequences, and the remaining sequences clustered at 95%, achieving a benchmark accuracy of 99.899%. Bowtie 2 (version 2.5.2) [93] was then used to align and remove reads derived from the host (human) genome, utilizing the GRCh38.p13 reference genome obtained from GenCode. The alignment was performed in the very-sensitive-local mode to maximize accuracy.

### Microbiome detection and quantification

The clean reads from each sample were annotated using Kraken2 (version 2.1.3) [94], a taxonomic classification system that employs a *k*-mer-based method to assign taxonomic labels quickly and accurately by matching *k*-mers to the lowest common ancestor of genomes. The PlusPF reference database, which includes combined annotations of archaeal, bacterial, viral, protozoal, and fungal sequences from the RefSeq databases, was used (downloaded on 2024 August 12). The confidence parameter was set as 0.1 for higher annotation accuracy. To enable intersample comparisons, microbial abundance was quantified as RPM. This was calculated by dividing the number of reads assigned to each taxon by the total number of reads in the library and multiplying the result by 10^6^. To minimize false positives, we applied stringent filtering criteria: bacterial taxa with an RPM below 10, and viral and fungal taxa with an RPM below 1, were excluded from further analysis. This filtering process was automated using a custom stand-alone script.

### Virus host curation

Full NCBI Virus metadata were obtained (2024 August 17) from the National Center for Biotechnology Information (NCBI) FTP site (https://ftp.ncbi.nlm.nih.gov/genomes/Viruses/AllNuclMetadata/AllNuclMetadata.csv.gz). The taxonomic lineage of each virus was manually curated using taxonkit (version 0.17.0) [95]. For each viral family, we curated the host range and the number of virus entries. Only viruses with hosts including “Human”, “Bacteria”, or “Fungi” were included for further analysis.

### Community diversity analysis

Alpha diversity was assessed to quantify microbial richness and diversity across samples. The analysis was conducted using the vegan R package (version 2.6-6.1) based on the filtered RPM table as mentioned above. For each sample, microbial richness was calculated as the number of taxa with nonzero abundance. Shannon and Gini–Simpson indices were used as the diversity measures, computed using the diversity function with the “shannon” method and the “simpson” method, respectively. For beta-diversity analysis, Bray–Curtis dissimilarity matrices were calculated at the genus, family, and species levels using the vegan package (version 2.6-6.1) in R. Principal coordinates analysis was then performed on the Bray–Curtis dissimilarity matrices, retaining the first 2 principal coordinates for visualization. Eigenvalues were computed to determine the proportion of variance explained by each coordinate.

### Pathogenic and nonpathogenic microorganism analysis

Metadata for human pathogens, including 510 bacterial taxa, 407 fungal taxa, and 226 viral taxa, were obtained from the gcPathogen database (downloaded on 2024 August 17, https://nmdc.cn/gcpathogen/) [49]. The taxonomic lineage of each pathogen was curated manually using taxonkit (version 0.17.0) [95]. Microbes identified from this study were classified as pathogenic or nonpathogenic at the genus and species levels according to the organized information. A genus was considered pathogenic if it contained at least one pathogenic species. The relative abundance, and detection rate, calculated as the number of patients positive for the taxon divided by the total number of individuals in each group, of pathogenic genera and species were then compared across groups.

### Correlation network analysis

Correlation networks between viruses and microbial taxa were constructed using igraph (2.0.3) and ggraph (version 2.2.1) in R. Spearman correlations between viral, bacterial, and fungal genera in sputum and swab samples were calculated with false-discovery-rate-adjusted *P* values (*P* ≤ 0.05). The top 10 bacterial or fungal interactions per virus were retained for network visualization.

### Viral genome assembly, abundance estimation, and sequence annotation

Viral contigs were assembled based on clean reads by using Megahit (version 1.2.9) [96] with default parameters. The assembled contigs were annotated via BLASTp against the nonredundant protein (NR) database (downloaded on 2023 October 23) by using DIAMOND (version 2.1.9.163) [97]. Only those with viral sequences as the top hit were retained. Contigs were further evaluated using CheckV (version 1.0.3) [98], and only complete, high-quality, and medium-quality contigs containing at least one viral gene were kept as potential viral fragments. To estimate viral abundance, reads were mapped to the assembled contigs using Bowtie 2 (version 2.5.4) [93], and the normalized relative abundance of each contig was calculated as RPM, as previously described.

### Phylogenetic analysis and contig annotation of specific viruses

Prodigal with default parameters was used to predict proteins from viral contigs. Protein functions were annotated using BLASTp (version 2.15.0+) [99] against the NR database and CD-search against the Conserved Domain Database (version 3.21) [100]. To select reference sequences for phylogenetic analysis, all replication proteins from *Redondoviridae* and RNA-dependent RNA polymerases from *Human coronavirus 229E* in the NCBI database were chosen. For *Vientovirus* and *Human coronavirus 229E*, the respective replication proteins and RNA-dependent RNA polymerases were selected. These proteins were aligned with reference sequences using MAFFT (version 7.525) [101] with default settings. Ambiguously aligned regions were removed using TrimAl (version 1.5) [102]. Phylogenetic trees were constructed using FastTree (version 2.1.11) [103] with the LG model and visualized in iTOL [104]. The viral genomic structure was visualized using Geneious Prime (version 2024.0.5), and conserved domains of the replication proteins were predicted with InterPro using default parameters.

### Functional annotation and differential pathway analysis

Functional profiling of microbial communities was conducted using HUMAnN 3.0 to annotate the functional expression of the microbiome based on MetaCyc pathway definitions on sputum and swab samples, respectively. Pathway abundances were normalized to copies per million units using the humann_renorm_table tool Differential abundance analysis of MetaCyc pathways between LC and NLC groups was performed using the Wilcoxon signed-rank test.

### Correlation between microbial diversity and clinical features

Spearman correlation analysis was conducted to assess associations between microbial species in sputum and swab samples and the clinical features of patients in LC and NLC groups. Correlations were calculated using the psych package (version 2.4.6.26), and significance was determined with false-discovery-rate-adjusted *P* values (*P* ≤ 0.05). Significant correlations were visualized using pheatmap (version 1.0.12). Heatmaps were generated for each group, and significant species correlations were highlighted by using scatter plots.

### Statistical analysis

Given the non-normal distribution of continuous variables in this study, as determined by normality testing, the Wilcoxon signed-rank test was used for group comparisons of numerical variables. The chi-square test was employed to assess differences in microbial detection rates across groups. Differences in microbial community composition between groups were evaluated using permutational multivariate ANOVA via the adonis2 function of the vegan package. Spearman rank correlation analysis was performed to assess correlations between microbial abundance and clinical characteristics. All statistical tests were 2-tailed, and *P* values <0.05 were considered statistically significant.

## Ethical Approval

Ethical approval for this study was granted by the Ethics Committee of Wuxi Fifth People’s Hospital (approval no. 2022-019-1). All procedures performed were in accordance with the ethical standards of the committee. Written informed consent was obtained from all participants prior to enrollment in the study.

## Data Availability

The raw sequence data generated in this paper have been deposited in the Genome Sequence Archive in the National Genomics Data Center, China National Center for Bioinformation/Beijing Institute of Genomics, Chinese Academy of Sciences (GSA: CRA020324), which are publicly accessible at https://ngdc.cncb.ac.cn/gsa [105,106]. This study did not generate new unique reagents.
