# Supplementary material for: Meta-transcriptomics Reveals Dysbiosis of the Respiratory Microbiome in Older Adults with Long COVID
Source: Research (Wash D C). 2025 Jun 2;8:0720. doi: 10.34133/research.0720 (PMC12128852; doi:10.34133/research.0720)
Supplement: Supplementary 1 — Figs. S1 to S6 Table S1 [file research.0720.f1.pdf]

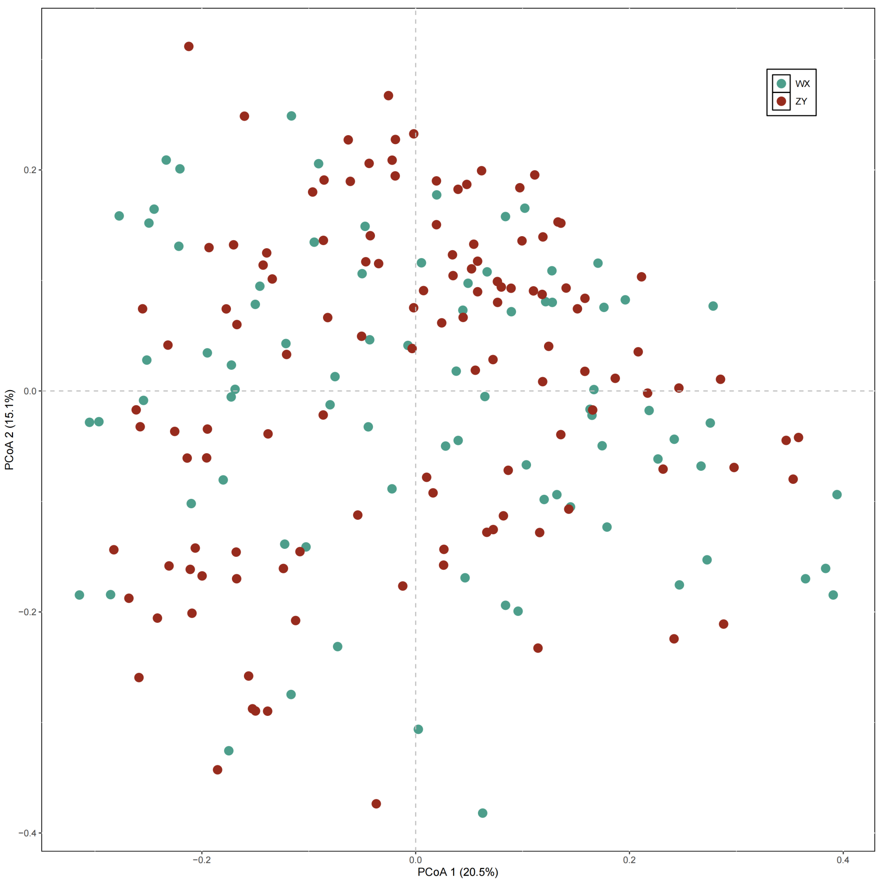

WX
ZY
PCoA 1 (20.5%)
PCoA 2 (15.1%)
−0.2
0.0
0.2
0.4
−0.4

Figure S1. Principal Coordinate Analysis (PCoA) based on Bray-Curtis dissimilarity illustrating difference of microbial community composition among two centers.

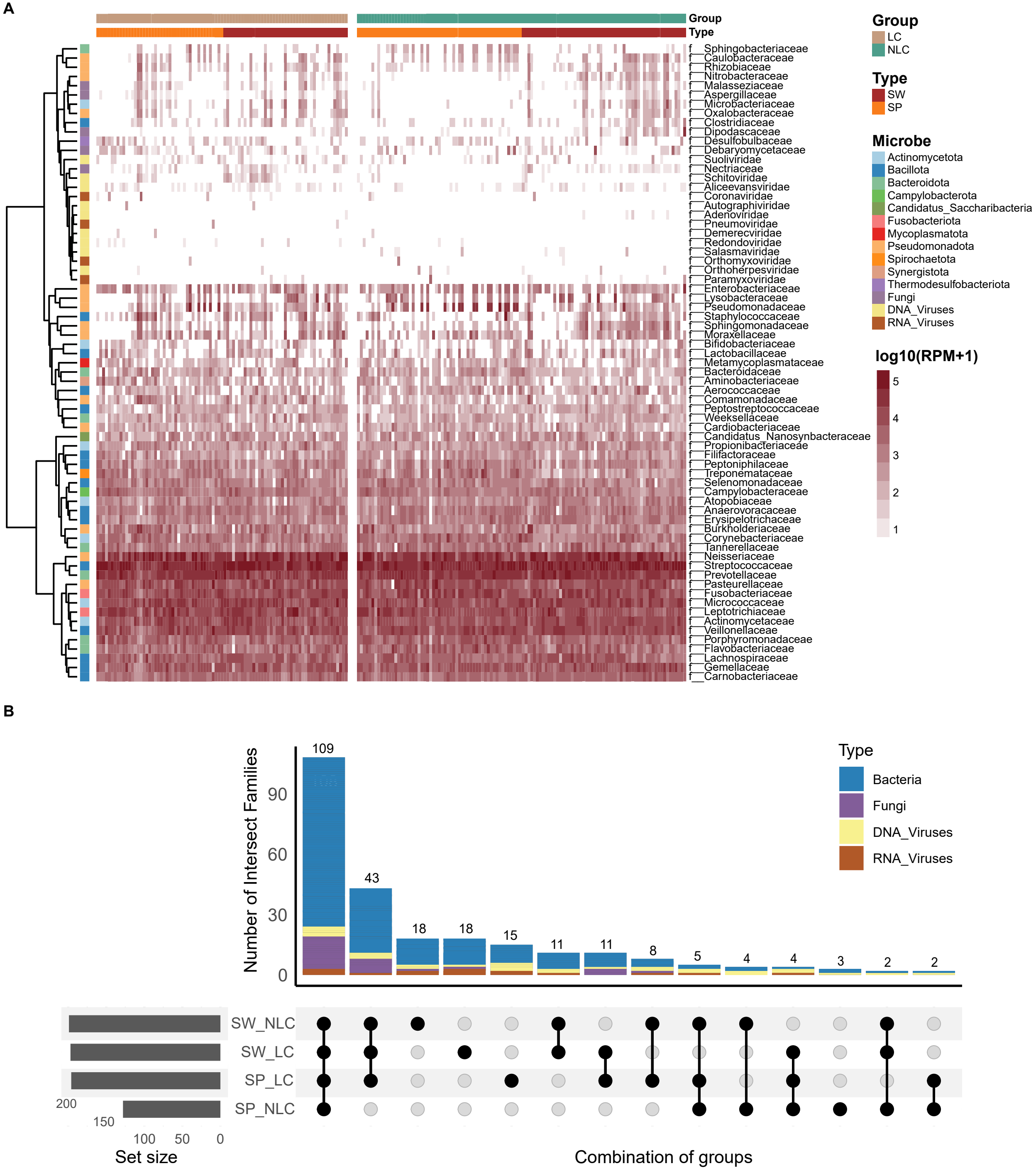

A
Group
Type
LC
NLC
SW
SP
Microbe
Actinomycetota
Bacillota
Bacteroidota
Campylobacterota
Candidatus_Saccharibacteria
Fusobacteriota
Mycoplasmatota
Pseudomonadota
Spirochaetota
Synergistota
Thermodesulfobacteriota
Fungi
DNA_Viruses
RNA_Viruses
log10(RPM+1)
5
4
3
2
1
f__Sphingobacteriaceae
f__Caulobacteraceae
f__Rhizobiaceae
f__Nitrobacteraceae
f__Malasseziaceae
f__Aspergillaceae
f__Microbacteriaceae
f__Oxalobacteraceae
f__Clostridiaceae
f__Dipodascaceae
f__Desulfobulbaceae
f__Debaryomycetaceae
f__Suoliviridae
f__Nectriaceae
f__Schitoviridae
f__Aliceevansviridae
f__Coronaviridae
f__Autographiviridae
f__Adenoviridae
f__Pneumoviridae
f__Demerecviridae
f__Redondoviridae
f__Salasmaviridae
f__Orthomyxoviridae
f__Orthoherpesviridae
f__Paramyxoviridae
f__Enterobacteriaceae
f__Lysobacteraceae
f__Pseudomonadaceae
f__Staphylococcaceae
f__Sphingomonadaceae
f__Moraxellaceae
f__Bifidobacteriaceae
f__Lactobacillaceae
f__Metamycoplasmataceae
f__Bacteroidaceae
f__Aminobacteriaceae
f__Aerococcaceae
f__Comamonadaceae
f__Peptostreptococcaceae
f__Weeksellaceae
f__Cardiobacteriaceae
f__Candidatus_Nanosynbacteraceae
f__Propionibacteriaceae
f__Filifactoraceae
f__Peptoniphilaceae
f__Treponemataceae
f__Selenomonadaceae
f__Campylobacteraceae
f__Atopobiaceae
f__Anaerovoracaceae
f__Erysipelotrichaceae
f__Burkholderiaceae
f__Corynebacteriaceae
f__Tannerellaceae
f__Neisseriaceae
f__Streptococcaceae
f__Prevotellaceae
f__Pasteurellaceae
f__Fusobacteriaceae
f__Micrococcaceae
f__Leptotrichiaceae
f__Actinomycetaceae
f__Veillonellaceae
f__Porphyromonadaceae
f__Flavobacteriaceae
f__Lachnospiraceae
f__Gemellaceae
f__Carnobacteriaceae
B
Type
Bacteria
Fungi
DNA_Viruses
RNA_Viruses
Number of Intersect Families
0
30
60
90
109
43
18
18
15
11
11
8
5
4
4
3
2
2
SW_NLC
SW_LC
SP_LC
SP_NLC
200
150
100
50
0
Set size
Combination of groups

Figure S2. Family level microbial composition

(A) Heatmap of family-level microbial composition across different sample types (SP and SW) and patient groups (LC and NLC). The log10-transformed RPM values of 110 microbial families are displayed, clustered based on their abundance patterns.

(B)Upset plot illustrating the distribution and overlap of microbial families across four sample groups.The horizontal bars indicate the set size for each group, while vertical bars represent the number of intersecting families for specific group combinations. The bars are color-coded by microbial type, including Bacteria, Fungi, DNA Viruses, and RNA Viruses. The connected black dots below the bars denote the sample group combinations contributing to each intersection.

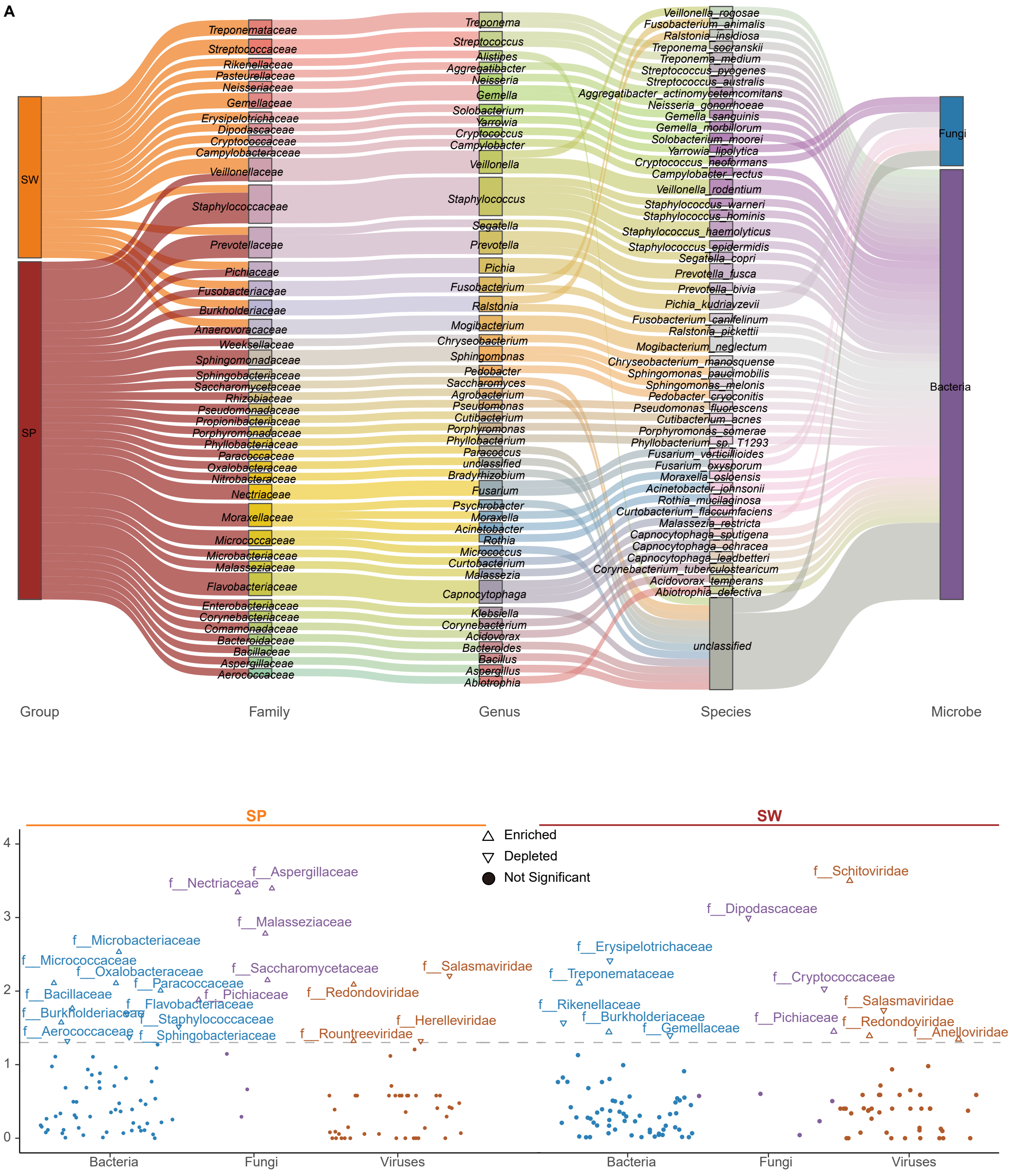
A
SW
SP
Treponemataceae
Streptococcaceae
Rikenellaceae
Pasteurellaceae
Neisseriaceae
Gemellaceae
Erysipelotrichaceae
Dipodascaceae
Cryptococcaceae
Campylobacteraceae
Veillonellaceae
Staphylococcaceae
Prevotellaceae
Pichiaceae
Fusobacteriaceae
Burkholderiaceae
Anaerovoracaceae
Weeksellaceae
Sphingomonadaceae
Sphingobacteriaceae
Saccharomycetaceae
Rhizobiaceae
Pseudomonadaceae
Propionibacteriaceae
Porphyromonadaceae
Phyllobacteriaceae
Paracoccaceae
Oxalobacteraceae
Nitrobacteraceae
Nectriaceae
Moraxellaceae
Micrococcaceae
Microbacteriaceae
Malasseziaceae
Flavobacteriaceae
Enterobacteriaceae
Corynebacteriaceae
Comamonadaceae
Bacteroidaceae
Bacillaceae
Aspergillaceae
Aerococcaceae
Treponema
Streptococcus
Alistipes
Aggregatibacter
Neisseria
Gemella
Solobacterium
Yarrowia
Cryptococcus
Campylobacter
Veillonella
Staphylococcus
Segatella
Prevotella
Pichia
Fusobacterium
Ralstonia
Mogibacterium
Chryseobacterium
Sphingomonas
Pedobacter
Saccharomyces
Agrobacterium
Pseudomonas
Cutibacterium
Porphyromonas
Phyllobacterium
Paracoccus
unclassified
Bradyrhizobium
Fusarium
Psychrobacter
Moraxella
Acinetobacter
Rothia
Micrococcus
Curtobacterium
Malassezia
Capnocytophaga
Klebsiella
Corynebacterium
Acidovorax
Bacteroides
Bacillus
Aspergillus
Abiotrophia
Veillonella_rogosae
Fusobacterium_animalis
Ralstonia_insidiosa
Treponema_socranskii
Treponema_medium
Streptococcus_pyogenes
Streptococcus_australis
Aggregatibacter_actinomycetemcomitans
Neisseria_gonorrhoeae
Gemella_sanguinis
Gemella_morbillorum
Solobacterium_moorei
Yarrowia_lipolytica
Cryptococcus_neoformans
Campylobacter_rectus
Veillonella_rodentium
Staphylococcus_warneri
Staphylococcus_hominis
Staphylococcus_haemolyticus
Staphylococcus_epidermidis
Segatella_copri
Prevotella_fusca
Prevotella_bivia
Pichia_kudriavzevii
Fusobacterium_canifelinum
Ralstonia_pickettii
Mogibacterium_neglectum
Chryseobacterium_manosquense
Sphingomonas_paucimobilis
Sphingomonas_melonis
Pedobacter_cryoconitis
Pseudomonas_fluorescens
Cutibacterium_acnes
Porphyromonas_somerae
Phyllobacterium_sp._T1293
Fusarium_verticillioides
Fusarium_oxysporum
Moraxella_osloensis
Acinetobacter_johnsonii
Rothia_mucilaginosa
Curtobacterium_flaccumfaciens
Malassezia_restricta
Capnocytophaga_sputigena
Capnocytophaga_ochracea
Capnocytophaga_leadbetteri
Corynebacterium_tuberculostearicum
Acidovorax_temperans
Abiotrophia_defectiva
unclassified
Fungi
Bacteria
Group
Family
Genus
Species
Microbe
B
SP
SW
Enriched
Depleted
Not Significant
−log10(P_value)
0
1
2
3
4
f__Aspergillaceae
f__Nectriaceae
f__Malasseziaceae
f__Microbacteriaceae
f__Micrococcaceae
f__Oxalobacteraceae
f__Saccharomycetaceae
f__Salasmaviridae
f__Paracoccaceae
f__Bacillaceae
f__Pichiaceae
f__Redondoviridae
f__Flavobacteriaceae
f__Burkholderiaceae
f__Staphylococcaceae
f__Herelleviridae
f__Aerococcaceae
f__Sphingobacteriaceae
f__Rountreeviridae
f__Schitoviridae
f__Dipodascaceae
f__Erysipelotrichaceae
f__Treponemataceae
f__Cryptococcaceae
f__Salasmaviridae
f__Rikenellaceae
f__Burkholderiaceae
f__Pichiaceae
f__Redondoviridae
f__Gemellaceae
f__Anelloviridae
Bacteria
Fungi
Viruses
Bacteria
Fungi
Viruses

Figure S3. Lineage and Classification of Microbes Identified from Swab (SW) and Sputum (SP) Samples

(A) The Sankey plot shows the hierarchical classification of pathogenic microbes from "Group" (SW or SP) through "Family," "Genus," and "Species," with microbes classified as fungi or bacteria on the far right. The width of the bands reflects the entries of each taxon across sample types.

(B) Manhattan plot depicting the significantly different microbial families between LC and NLC groups in SP (left) and SW (right) samples. Bacteria (blue), fungi (purple), and viruses (orange) are displayed, with family names labeled for significantly enriched or depleted taxa in LC. Upward triangles indicate LC-enriched families, while downward triangles represent LC-depleted families.

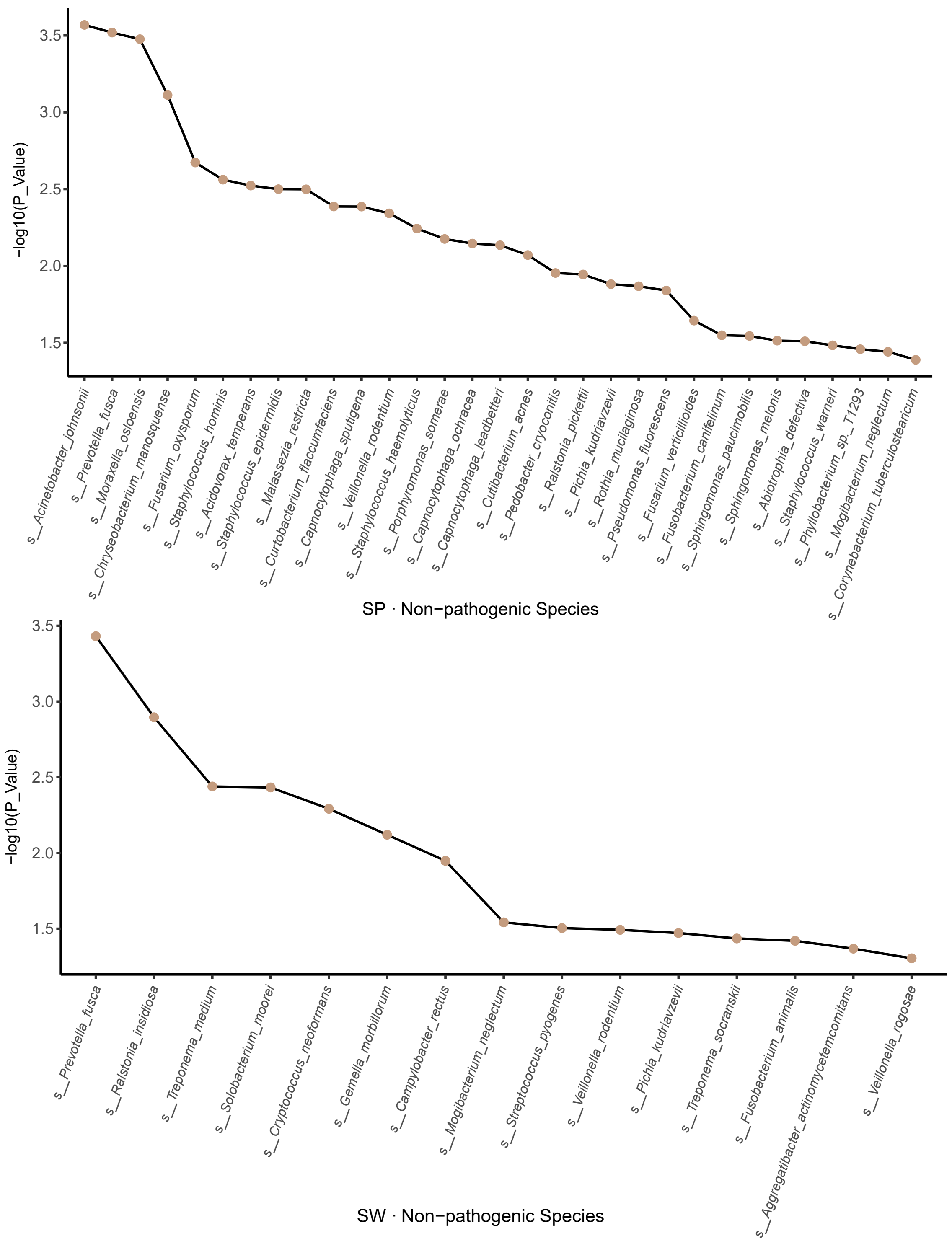

3.5
3.0
2.5
2.0
1.5
-log10(P_Value)
s__Acinetobacter_johnsonii
s__Prevotella_fusca
s__Moraxella_osloensis
s__Chryseobacterium_manosquense
s__Fusarium_oxysporum
s__Staphylococcus_hominis
s__Acidovorax_temperans
s__Staphylococcus_epidermidis
s__Malassezia_restricta
s__Curtobacterium_flaccumfaciens
s__Capnocytophaga_sputigena
s__Veillonella_rodentium
s__Staphylococcus_haemolyticus
s__Porphyromonas_somerae
s__Capnocytophaga_ochracea
s__Capnocytophaga_leadbetteri
s__Cutibacterium_acnes
s__Pedobacter_cryoconitis
s__Ralstonia_pickettii
s__Pichia_kudriavzevii
s__Rothia_mucilaginosa
s__Pseudomonas_fluorescens
s__Fusarium_verticillioides
s__Fusobacterium_canifelinum
s__Sphingomonas_paucimobilis
s__Sphingomonas_melonis
s__Abiotrophia_defectiva
s__Staphylococcus_warneri
s__Phyllobacterium_sp._T1293
s__Mogibacterium_neglectum
s__Corynebacterium_tuberculostearicum
SP · Non−pathogenic Species
3.5
3.0
2.5
2.0
1.5
-log10(P_Value)
s__Prevotella_fusca
s__Ralstonia_insidiosa
s__Treponema_medium
s__Solobacterium_moorei
s__Cryptococcus_neoformans
s__Gemella_morbillorum
s__Campylobacter_rectus
s__Mogibacterium_neglectum
s__Streptococcus_pyogenes
s__Veillonella_rodentium
s__Pichia_kudriavzevii
s__Treponema_socranskii
s__Fusobacterium_animalis
s__Aggregatibacter_actinomycetemcomitans
s__Veillonella_rogosae
SW · Non−pathogenic Species

Figure S4. Differential Abundance of Non-Pathogenic Microbial Species in SP and SW Samples

Line plots showing non-pathogenic microbial species with significant differences in relative abundance between SP (top) and SW (bottom) sample groups.

The x-axis lists microbial species ranked by significance, and the y-axis represents the −log10(P-value) for each species.

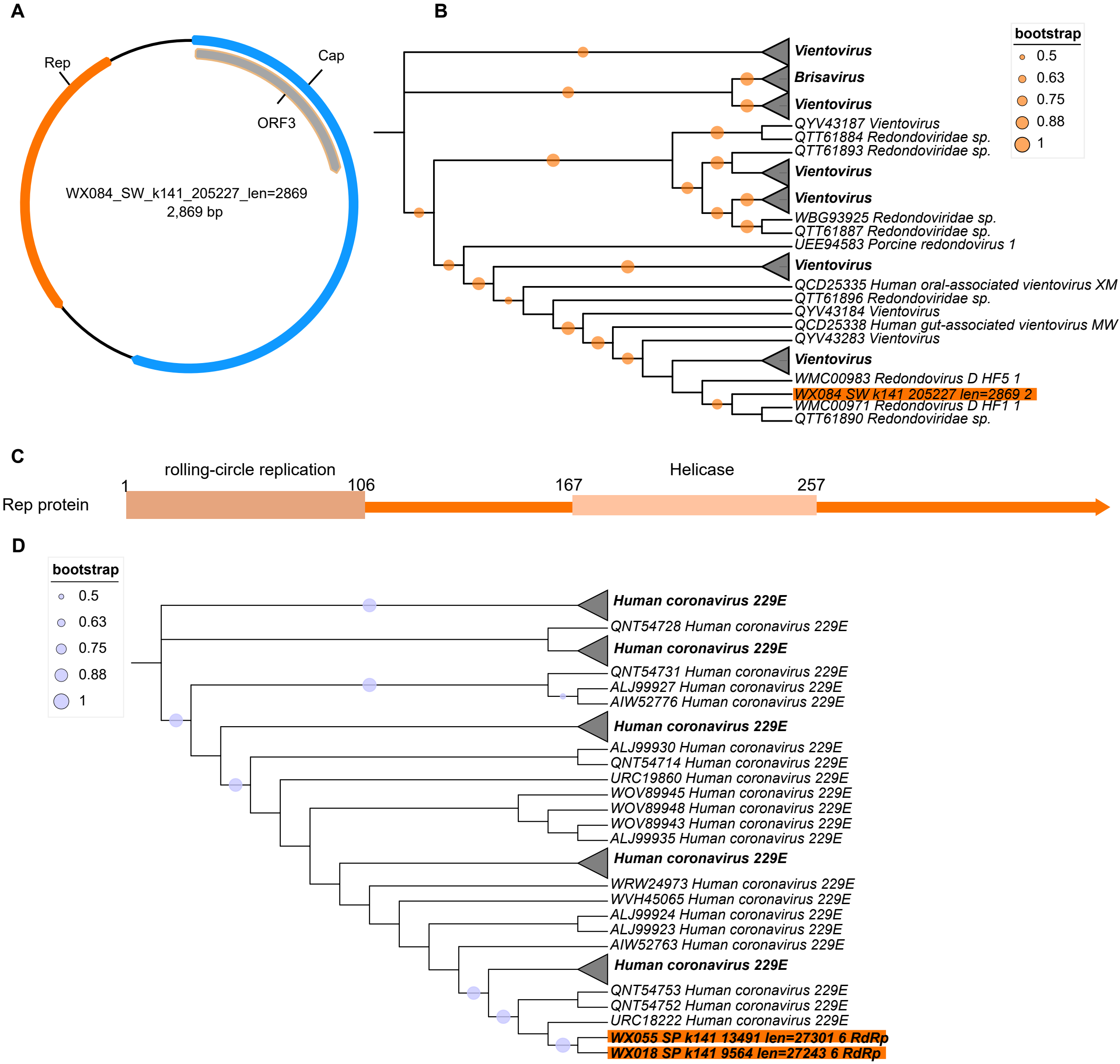
A
Rep
Cap
ORF3
WX084_SW_k141_205227_len=2869
2,869 bp
B
bootstrap
0.5
0.63
0.75
0.88
1
Vientovirus
Brisavirus
Vientovirus
QYV43187 Vientovirus
QTT61884 Redondoviridae sp.
QTT61893 Redondoviridae sp.
Vientovirus
Vientovirus
WBG93925 Redondoviridae sp.
QTT61887 Redondoviridae sp.
UEE94583 Porcine redondovirus 1
Vientovirus
QCD25335 Human oral-associated vientovirus XM
QTT61896 Redondoviridae sp.
QYV43184 Vientovirus
QCD25338 Human gut-associated vientovirus MW
QYV43283 Vientovirus
Vientovirus
WMC00983 Redondovirus D HF5 1
WX084 SW k141 205227 len=2869 2
WMC00971 Redondovirus D HF1 1
QTT61890 Redondoviridae sp.
C
rolling-circle replication
Helicase
1
106
167
257
Rep protein
D
bootstrap
0.5
0.63
0.75
0.88
1
Human coronavirus 229E
QNT54728 Human coronavirus 229E
Human coronavirus 229E
QNT54731 Human coronavirus 229E
ALJ99927 Human coronavirus 229E
AIW52776 Human coronavirus 229E
Human coronavirus 229E
ALJ99930 Human coronavirus 229E
QNT54714 Human coronavirus 229E
URC19860 Human coronavirus 229E
WOV89945 Human coronavirus 229E
WOV89948 Human coronavirus 229E
WOV89943 Human coronavirus 229E
ALJ99935 Human coronavirus 229E
Human coronavirus 229E
WRW24973 Human coronavirus 229E
WVH45065 Human coronavirus 229E
ALJ99924 Human coronavirus 229E
ALJ99923 Human coronavirus 229E
AIW52763 Human coronavirus 229E
Human coronavirus 229E
QNT54753 Human coronavirus 229E
QNT54752 Human coronavirus 229E
URC18222 Human coronavirus 229E
WX055 SP k141 13491 len=27301 6 RdRp
WX018 SP k141 9564 len=27243 6 RdRp

Figure S5. Genomic Structure and Phylogenetic Analysis of Vientovirus sp. and Human Coronavirus 229E.

(A) Circular genome map of Vientovirus sp., showing the presence of a replication-associated protein (Rep), capsid protein (Cap), and an open reading frame 3 (ORF3).

(B) Phylogenetic tree of replication proteins from Vientovirus and related Redondoviridae members. Bootstrap values are represented by circle size.

(C) Domain structure of the Vientovirus Rep protein, with annotated rolling-circle replication and helicase domains.

(D) Phylogenetic tree of RNA-dependent RNA polymerase (RdRp) proteins from Human Coronavirus 229E, including closely related strains. Bootstrap support values are shown by circles of varying sizes. Key viral contigs identified in this study are highlighted in orange.

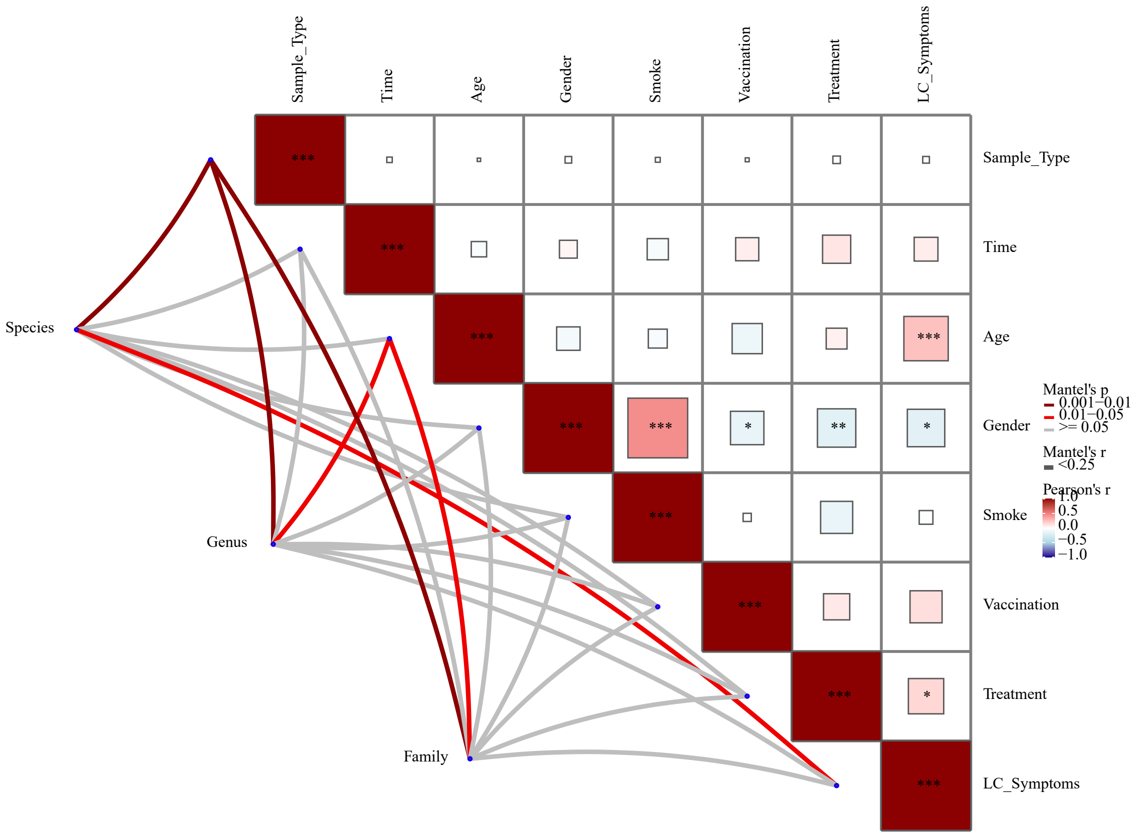
Sample_Type
Time
Age
Gender
Smoke
Vaccination
Treatment
LC_Symptoms
Species
Genus
Family
Mantel's p
0.001−0.01
0.01−0.05
>= 0.05
Mantel's r
<0.25
Pearson's r
1.0
0.5
0.0
−0.5
−1.0

Figure S6. Correlation of Microbial Diversity with Patient Characteristics.

Heatmap and network visualization showing correlations between microbial diversity (species, genus, family), sample type and clinical factors (age, gender, smoking, vaccination, treatment, LC symptoms). Significant Pearson correlations are marked with asterisks (*$P < 0.05$, **$P < 0.01$, ***$P < 0.001$). Network lines represent Mantel's test results, with thickness indicating the correlation coefficient and color denoting significance.

Supplementary Table 1. Characteristics of patients included in this study

Characteristics	LC Group (n=44)	NLC Group (n=57)
**Age, Ave. ± STD**	65.2 ± 8.3	60.8 ± 10.2
**Gender, n (%)**		
Male	20 (45.5%)	35 (61.4%)
Female	24 (54.5%)	22 (38.6%)
**Times of Infection, n (%)**		
1	22 (50%)	33 (57.9%)
2	17 (38.6%)	18 (31.6%)
3	4 (9.1%)	1 (1.8%)
4	0 (0%)	1 (1.8%)
**Vaccination status, n (%)**		
0	4 (9.1%)	9 (15.8%)
1	0 (0%)	3 (5.3%)
2	8 (18.2%)	10 (17.5%)
3	31 (70.5%)	33 (57.9%)
4	1 (2.3%)	2 (3.5%)
**Smoke, n (%)**		
Yes	11 (25.0%)	15 (26.3%)
No	33 (75.0%)	42 (73.7%)
**Treatment during infection, n (%)**		
Yes	19 (43.2%)	15 (26.3%)
No	25 (56.8%)	42 (73.7%)
**LC symptoms, n**		
Non-specific	31	
Cardiopulmonary	23	
Neurological	11	
Gastrointestinal	7	
Upper Respiratory	10	
Skeletal/muscle	6	

Note: LC: Long COVID; NLC: Non-Long COVID. Age is presented as the average (Ave) ± standard deviation (STD). Age group, gender, times of infection, vaccination

status, smoke, and treatment are represented as the number of patients (n) and the percentage (%) of the total group. LC symptoms are listed by category, with the corresponding number of patients in each category.
